# Start-Ups Are Supercharging Algae to Clean Up the Environment

**DOI:** 10.1021/acscentsci.4c01599

**Published:** 2024-10-16

**Authors:** Avya Chaudhary

On the barren coast of Akhfenir, Morocco, a nature-based solution to the climate crisis is taking shape. Raffael Jovine is building an oblong, mechanically stirred pool full of pea-green water. He claims it’s the world’s largest “algal raceway pond.” Bordered by desert on one side and cold ocean currents on the other, this seemingly inhospitable land is where Jovine found the perfect algal strains to capture carbon dioxide.

**Figure:**
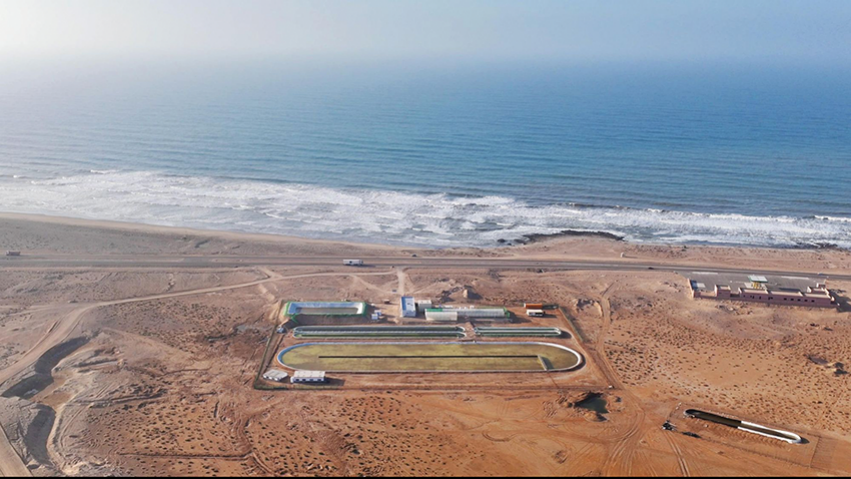
At Brilliant Planet’s algae farm in Akhfenir, Morocco, the team uses local algal strains to sequester carbon and deacidify the ocean. Credit: Brilliant Planet.

Jovine, the cofounder and chief scientist of Brilliant Planet, is trying to engineer high-growth microalgae strains to capture and bury carbon as biomass in the ground for 1,000 years. In August 2023, the company achieved a breakthrough by growing enough algae to fill 77 Olympic-size swimming pools in just a month.

“These locally sourced marine organisms are fast growers even in extreme conditions,” Jovine says. “They grow like weeds and can sequester up to 50 times more carbon dioxide from the atmosphere per hectare per year than a typical temperate forest.”

After incorporating carbon, these engineered algae are propelled to the top of a 10-story tower. Once sprayed into the desert air, the biomass dries into a flurry of hypersaline flakes in less than 30 s. The flakes are collected and buried underground, entombing any carbon they’ve consumed.

This is more than an exercise in sustainability. Brilliant Planet’s facility generates carbon credits that the company has already sold to the tech giant Block to offset 1,500 metric tons of carbon dioxide by 2027.

Jovine isn’t alone in believing that algae hold the key to our climate future. He’s part of a growing wave of start-ups that see algae as a multipronged solution for capturing carbon dioxide, mopping up pollution, and cleaning water supplies. Funding for these companies is mounting, but they still have to find ways to work around natural algae’s biological and commercial limitations. Using genetic engineering and advanced growth techniques, they’re creating high-quality algal strains that push past these obstacles.

## Algae for carbon capture

What makes algae so attractive is their biochemistry. They can grow 10 times as fast as terrestrial plants, produce 70% of Earth’s oxygen, and capture up to 2.5 tons of carbon dioxide per acre daily. But making a business out of algae isn’t as simple as it sounds. Their short lifespan, dietary needs, and susceptibility to contamination make turning a profit on algae products difficult.

For example, Jovine says, algae can be so finicky that it’s not uncommon for them to fail after 12 min in field tests for biofuel production. The reason for that is “outdated algal strains that have been sitting on the university shelf for the last 120 years,” he says.

Rather than use established strains that have been adapted to grow in a lab under “dingy lights,” as Jovine puts it, Brilliant Planet starts with algae native to Morocco and grows them in the open air. The company then optimizes culture parameters like temperature and light, as well as individual nutrients such as nitrogen, silicate, and iron, to coax cells to grow faster and capture more CO_2_ than they could naturally.

To get its operation working, Jovine’s team had to devise a way to keep pH levels in its ponds in check. When the algae pond is filled with ocean water, it begins with an acidic pH, a product of CO_2_ emissions in the air dissolving into the ocean and turning into carbonic acid. As the algae gobble up those acidic compounds, the water’s pH starts to rise. That shift creates carbonate, which the algae can’t use to grow. To avoid carbonate production, Brilliant Planet injects more CO_2_ into the pond, feeds the algae a diet that acidifies their water, and raises or lowers the system’s stirring speed.

Dialing in these parameters allows algae to grow 300,000 cells per milliliter, up from their natural density of 20,000–30,000. In addition, Brilliant Planet’s technology has a knock-on effect of deacidifying the ocean. After the water runs through the algae pond, the company returns the water to the ocean without the carbonic acid. The facility neutralizes so much acid that “for every unit of ocean water we bring in, we deacidify about 5.1 units,” Jovine says.

Brilliant Planet is scaling up its project faster than many efforts for direct air capture (DAC) of CO_2_ have managed. For one, algae-based projects have the benefit of deriving much of their energy inputs from the sun. But another big part of their success comes from circumventing the energetic penalty that comes with collecting scattered carbon dioxide molecules from the environment: DAC tries to nab CO_2_ molecules floating in the air and stuff them into a solid sorbent; corralling and holding on to all those molecules requires a significant energy input.

Algae skip this expensive energy trap because their photosynthetic cells use the sun’s energy to actively transport CO_2_ or bicarbonate across their cellular membranes. Overall, algae-based carbon capture can cost 20–25% as much as DAC.

Backed by more than $25 million from companies including Toyota Ventures and Pegasus Tech Ventures, Brilliant Planet has completed its pilot project and is moving to build a 70,000 m^2^ demonstration site that will be able to slurp up 100 tons of CO_2_ annually by early 2025. If all goes according to plan, the firm aims to expand this operation into a 14 million m^2^ commercial facility designed to capture 270,000 tons of CO_2_ per year by 2030.

## Genetically engineering algae to trap more carbon

Other start-ups have goals similar to those of Brilliant Planet but take a more fundamental approach: genetic engineering. At The Oxford Science Park, CyanoCapture uses altered algal genomes to grow high-metabolism strains of *Synechococcus*, a genus of cyanobacteria, better known as blue-green algae.

The start-up employs CRISPR-Cas12a to edit genes tied to carbon-fixing pathways that redirect carbon and use excess electrons created in the chloroplast. One of CyanoCapture’s approaches involves using excess electrons created during photosynthesis more efficiently. The start-up does this by splicing in more genetic pathways that can take advantage of the surplus chemical energy.

According to Stuart Reid, chief technology officer at CyanoCapture, the results are impressive. Their strains capture carbon dioxide twice as fast as a model cyanobacteria strain and three times as fast as other algal strains.

They’re energy efficient as well. While methods like DAC require 2,000 kW h of electricity per ton of CO_2_ captured, CyanoCapture’s strains work with a mere 350 kW h. They’re essentially self-sufficient and easily replicate themselves, which minimizes operating costs.

Even though CyanoCapture is still in the pilot phase, it has caught global attention, snagging the Shell New Energy Challenge award, an XPrize grant from the Musk Foundation, and grants from Innovate UK.

A similar effort is taking place in Hong Kong, where rows upon rows of photobioreactors (PBRs) fill ALcarbo Technology’s facility, each of them teeming with green algae and a tailored supply of CO_2_ and essential nutrients. These are the breeding grounds for the start-up’s genetically engineered algae that guzzle carbon dioxide at 12 times the fixation rate of wild-type strains.

The process begins by exposing natural strains of algae to chemical agents and other forms of environmental stressors that induce random mutations in their DNA. This creates a diverse pool of mutants, some of which may have enhanced carbon-fixation abilities. Inside the PBRs, conditions are meticulously controlled to push algae to their limits, maximizing mutation rates while ensuring they can survive and multiply.

**Figure:**
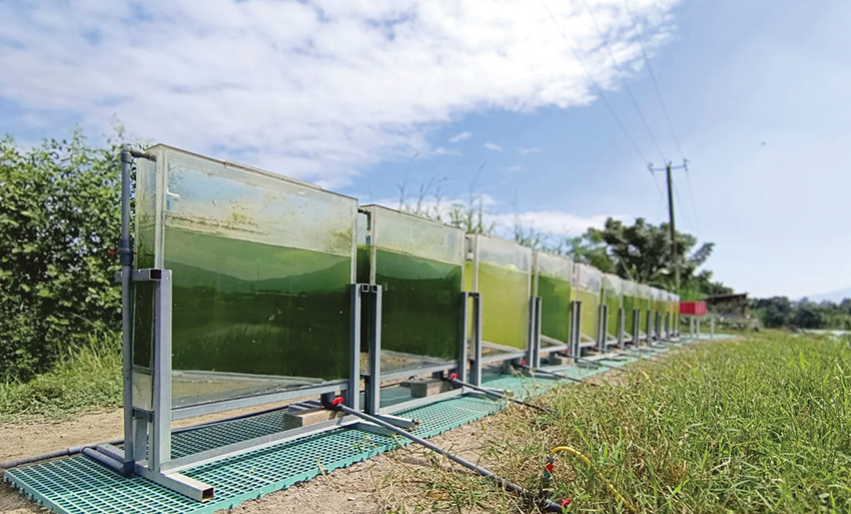
ALcarbo’s facility in Hong Kong is developing genetically engineered algal strains for carbon fixation. Credit: ALcarbo Technologies.

Besides genetic mutation, a critical component of ALcarbo’s PBRs are gas bubbles less than 200 nm in diameter dispersed throughout the water in which the algae grow. These nanobubbles contain and enhance the dissolution of gases like CO_2_ and oxygen to expedite the growth of mutated strains.

ALcarbo then selects the best-performing mutants based on visual traits like growth rate, color, and overall health. The most promising candidates undergo further analysis to home in on the precise genetic features that contribute to the mutants’ superior carbon-fixation abilities. Once those genes are documented, the process begins anew using the top-performing mutants.

“Even after accounting for the energy used to run and build them, these reactors can still [each] absorb half a ton of carbon dioxide each year,” says Nelson Ng, a cofounder of ALcarbo. The project is in its pilot phase, with 12 photobioreactors installed across 150 m^2^ in Yuen Long, Hong Kong. The company hopes to capture 100 tons of CO_2_ per year by next year, which will mean scaling up to over 2,900 bioreactors on an area about half the size of a football field.

In a world that rewards such research, mitigating climate change is a high-profile and potentially profitable goal, but algae’s environmental applications don’t stop there. The same principle—having algae wolf down molecules that they use to grow—can also be applied to cleaning up water.

## Soaking up pollution

“Water has often been overlooked in the environmental movement, like the Cinderella of the cause,” says Mahshid Sedghi, research and development director at the start-up Algaesys. “Many economies take it for granted, but there’s a growing disparity in access to clean water.”

At the literal center of the Portugal-based company’s technology are a series of rotating logs, hypnotic as they churn the water running through a trough beneath them. Each roller sports a carpet of growing algae that nabs pollutants each time it dips into the water.

**Figure:**
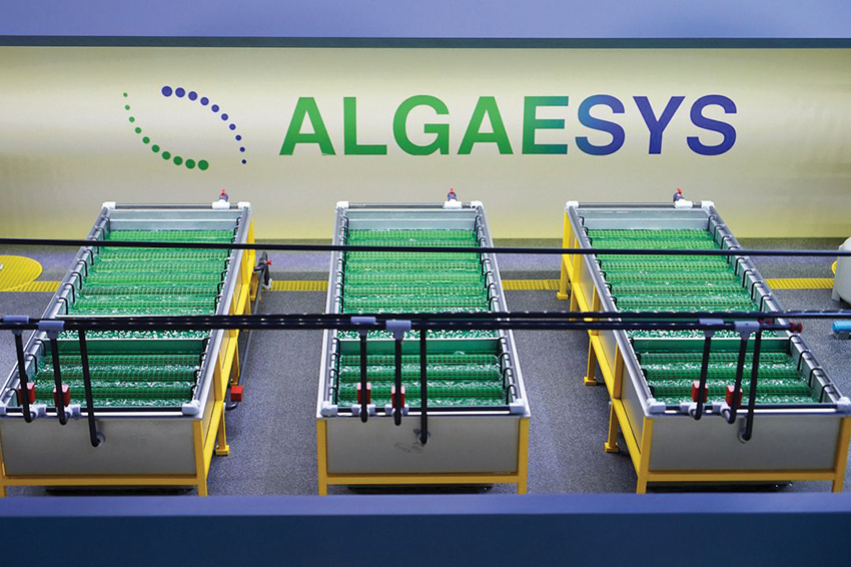
Algaesys’s rotating log system uses algae biofilm for wastewater treatment. Credit: Algaesys.

The algae naturally release sticky polymeric substances that serve as a foundation for biofilms, structured communities of microorganisms. The biofilms clean the water by generating reactive oxygen species that oxidize and break down complex pollutants, including microplastics and heavy metals coming from urban sewage, chemical sites, microelectronics, and industrial parks.

Notably, Sedghi says, Algaesys’s troughs can be deployed at various scales. While the company’s treatment plants in the US, the UK, China, and Australia are powered by large troughs and rollers, the system can be downsized to fit onto trucks for deployment in remote or low-resource areas.

“Our algae-based treatment is designed to address these issues on any scale—whether it’s a domestic rooftop setup or a massive facility handling 100 megaliters of wastewater a day,” Sedghi says. “This way, we can make water management equitable for everyone.”

The ingenuity of cleanup systems like this is that some wastewater pollutants actually feed plant cells. For example, runoff from farms that is rich in fertilizer chemicals such as ammonium, nitrate, and phosphorus-containing ions spurs growth in algae reactors.

Take as an example Archimede Ricerche’s facility in Camporosso, Italy. The 11,000 m^2^ glass building looks like a massive greenhouse full of rippling pools of water; sleek, cylindrical PBRs; and billions of microscopic algae that sustain themselves on nitrogen-contaminated wastewater coming into the plant from the food, beverage, and aquaculture industries.

When the wastewater enters the PBRs, the algae get to work, absorbing ammonium and nitrate through their cell membranes and using those nutrients to reproduce and renew their ranks.

Once the algae have done their job, the water emerges from the reactor cleaner and ready to be reused for numerous applications, including to irrigate nonedible crops or as an industrial coolant. As of now, Archimede’s treatment facility saves 50–70% in energy costs for wastewater treatment compared with a standard plant.

## Challenges and risks

Algae aren’t a silver bullet for the world’s environmental ills. For one thing, these projects still need to figure out what to do with the mounting mass of material they grow.

While Brilliant Planet buries the algae it sucks out of the ocean and sells off carbon credits, CyanoCapture uses algal strains to absorb and directly convert carbon into compounds of interest. “Imagine a power plant in Indonesia needing palmitic acid to replace palm oil in supply chains, or a company in Texas wanting graphite,” Reid says. “We can tailor our algae to produce exactly what they need.”

Another option is eating the stuff. Archimede is already selling biomass from its treatment plants as fish feed and as an ingredient in natural cosmetics. Its future plans include developing nutraceuticals for humans.

As these start-ups try to manipulate and generate profits from these organisms, they’re realizing that information on algae is scarce. The first genome database dedicated to algae was reported only in 2020 (Database, DOI: 10.1093/database/baaa097) and covers a small fraction of the algal world: among the 46,000-plus recognized species, the database contains information on fewer than 2,000.

“While we’ve been fermenting yeast for 5,000 years, these new and quirky microbes are often poorly understood,” Reid says. “We’re constantly in flux to find new ways to examine their genetic makeup and make better decisions about cultivation.”

In devising new ways to capture carbon dioxide, “we’re caught between tree planting and costly air capture technologies,” Jovine says. “We need to make algae a financial and social proposition that people can get behind. They’re a natural fix for human-made issues.”

## Avya Chaudhary is a freelance contributor to

Chemical & Engineering News, *the independent news outlet of the American Chemical Society*.

